# An In-Home Medication Dispensing System to Support Medication Adherence for Patients With Chronic Conditions in the Community Setting: Prospective Observational Pilot Study

**DOI:** 10.2196/34906

**Published:** 2022-05-19

**Authors:** Tejal Patel, Jessica Ivo, Teresa Pitre, Sadaf Faisal, Kristen Antunes, Kasumi Oda

**Affiliations:** 1 School of Pharmacy University of Waterloo Kitchener, ON Canada; 2 Centre for Family Medicine Family Health Team Kitchener, ON Canada; 3 Pack4U Toronto, ON Canada; 4 Catalyst Healthcare Kelowna, BC Canada

**Keywords:** smart, medication adherence, usability, geriatric, in-home, community, chronic diseases, medication dispensing, eHealth, platform, self management, support tool, chronic disease, caregiver, usability, satisfaction

## Abstract

**Background:**

Innovative digital technology systems that support and monitor real-time medication intake are now available commercially; however, there is limited knowledge of the use of such technology in patients’ homes. One such smart medication dispenser, *spencer*, provides alerts to patients to take their medications and allows for tracking and reporting real-time medication adherence data.

**Objective:**

The objectives of this study were to examine the use of a smart medication dispenser as a medication adherence and self-management support tool for community dwelling adults over a 6-month period, in addition to usability, usefulness, satisfaction, and impact on caregiver support.

**Methods:**

This prospective, observational study invited community-dwelling adults aged 45 years and older taking at least one chronic medication and their caregivers to use this smart medication dispenser for their medication administration for 6 months. Adherence was defined as a dose intake within 2 hours post scheduled time. Real-time adherence data were collected using the smart medication dispenser and the AdhereNet platform. Usability, usefulness, and satisfaction were measured using the System Usability Scale and the Usefulness, Satisfaction, and Ease of Use questionnaire, respectively. Caregiver burden was measured on a visual analog scale at baseline and at the end of the 6-month study period.

**Results:**

A total of 58 participants were recruited, of which 55% (32/58) were female with a mean age of 66.36 (SD 11.28; range 48-90) years. Eleven caregiver participants were recruited, of whom 91% (10/11) were female. The average monthly adherence over 6 months was 98% (SD 3.1%; range 76.5%-100%). The average System Usability score was 85.74 (n=47; SD 12.7; range 47.5-100). Of the 46 participants who provided data, 44 (96%) rated the product as easy, 43 (93%) as simple to use, and 43 (93%) were satisfied with the product. Caregiver burden prior to and following smart medication dispenser use for 6 months was found to be statistically significantly different (*P<*.001; CI 2.11-5.98).

**Conclusions:**

Smart medication adherence products such as *spencer*, when connected and clinically monitored, can be a useful solution for medication management and have the potential to improve caregiver burden.

## Introduction

Nonadherence to medications is a well-recognized global challenge. In 2003, the World Health Organization (WHO) noted that the mean adherence to chronic therapy was only 50% in high-income countries and even lower in low-income countries [1]. Numerous other studies confirm findings of significant nonadherence rates in various chronic disease populations [2-5]. Nonadherence to medications is a key contributor to potentially preventable health care utilization and costs [6-8]. Conversely, improving medication adherence can improve clinical outcomes, decrease mortality, and lower health care costs [9,10]. Several strategies to improve medication adherence have been identified, including patient education, medication regimen management, pharmacist-led interventions, cognitive behavioral therapies, medication-taking reminders, and incentivization [11]. Systematic reviews of several interventions indicate that while some interventions are effective [12,13] at improving medication adherence, others are not [14], and many are limited by the quality of studies conducted. Indeed, medication nonadherence continues to be a challenge and has led to an increasing interest in developing innovative digital technology systems that tackle medication taking and monitoring [15,16]. Some of these technologies include smart pill containers and wearable sensors that track medication access via actions such as opening containers, pouring pills, picking up pills, hand to mouth movements, or pill swallowing, while ingestible sensors detect medication ingestion [15]. Some systems, such as Medication Event Monitoring Systems, track and store the dates and times a vial is opened by simply incorporating a cap that can be fitted over prescription vials [17], while others offer multidose packaging, medication reminders via integrated alarms, text messaging, and notifications, among other things, when tracking medication adherence in real time [18]. However, the use of these smart medication dispensing aids in patients’ homes has not been investigated extensively.

While medication dispensing events that are tracked by dispensing aids have been validated as an accurate marker of medication adherence [11], there are several factors that may impact the implementation of such devices in the homes of patients. A key driver of the implementation and sustainable use of a product is its usability. The International Organization of Standardization defines usability as the “extent to which a system, product or service can be used by specified users to achieve a specified goal with effectiveness, efficiency and satisfaction in a specified context of use” [19]. As the definition indicates, medication dispensing devices must be established as effective, efficient, and satisfactory among the individuals expected to use the device. Complexity, design quality, and packaging can affect the implementation of eHealth products and should also be examined [20]. Unfortunately, few studies have examined the usability of such dispensing devices [21-24].

Older adults often require assistance with activities of daily living. Research indicates that informal caregivers, often spouses and children, provide the bulk of these services and that medication management is a key component of the health care assistance provided [25-27]. Caregivers perform a number of tasks associated with managing medications, including ordering and administering medications as well as monitoring adverse effects and the safe use of medications [28]. Medication management can be a cause of stress for caregivers. In addition to administration procedures and safety issues associated with medications, scheduling logistics such as administration of medication into care routines, scheduling multiple medications, giving medications on time, and keeping medication prescriptions filled are contributors to the burden a caregiver may experience. The use of medication reminder systems has been associated with a decrease in stress among caregivers of patients with dementia but not among those whose care recipients did not have dementia [29]. The study, however, did not specify the types of medication reminder systems that were used.

Therefore, the objectives of this study were to examine the use of one such system as a medication adherence and self-management support tool for adults in the community setting. In addition to examining the adherence to medication regimens during a 6-month period, we sought to examine the usability, usefulness, satisfaction, and impact on caregiver support. Finally, we examined the potential for pharmacists to identify and address medication related problems including concerns related to adherence with data available through AdhereNet.

## Methods

### Study Design

This study was designed as a prospective, observational pilot study.

### Sample and Sample Size

A convenience sample of 50 adults and 15 caregivers was determined as adequate for this pilot study. Patient participants had to be at least 45 years old, speak English, be a resident of Ontario, Canada, be prescribed at least one chronic oral medication, and have the cognitive capacity to interact with the medication dispensing device. Chronic oral medications were defined as a prescription or over-the-counter medication with approved indication or generally accepted reasons for use as defined by the WHO Anatomical Therapeutic Chemical/Defined Daily Dose Index 2019. Cognitive capacity to use the smart medication dispenser was determined by the research pharmacist using clinical judgment and by assessing responses to the following questions: was the participant comfortable opening the medication package? Was the participant able to tell how they could remove medications from the smart medication dispenser? Was the participant able to play back demonstration videos? Was the participant aware and able to call the number if there were any technical difficulties? Was the participant aware of the number to call if they needed the pharmacy’s assistance? If the respondent was not able to demonstrate capacity in the any of the above, they were excluded from participation.

Patient participants could have an unlimited number of scheduled medication dosing times, customized to their daily routines. All medications for a scheduled dosing time were packaged together in single or multiple multidose pouches. Adults who had previously expressed an interest in learning about the smart medication dispenser were approached to take part in this study. In addition, health care providers, outreach programs, and independent living communities were approached to assist in identifying potential participants. The participants’ caregivers were also invited to take part in this study. Caregiver participants comprised of family members, friends, volunteers, or paid support workers who had regular involvement with providing medication management support to the participants. Patient participants who were receiving assistance from a formal medication management program and those who had severe cognitive impairment were excluded from this study.

### Smart Medication Dispensing Device

In this study, we investigated the use of *spencer*, [30] an at-home smart medication dispensing device that connects patients to community pharmacists to monitor medication adherence, ask active engagement questions for patient reported outcomes, link readings from Bluetooth enabled devices to medication administration, and use telehealth capabilities with an embedded camera. Medications are packaged in multidose pouches by pharmacies certified on Catalyst Healthcare’s AdhereNet platform, [31] and delivered to patients at home weekly or biweekly. These multidose pouches are loaded into the smart medication dispenser at home and dispensed at individualized, appropriate preset times. The smart medication dispenser has a touch screen interface for patients to respond to reminder alerts, answer questions posed by their clinicians through the product, and participate in telehealth video calling through the device. The smart medication dispenser tracks a patient’s medication intake in real time and provides reports on adherence to the patient’s clinicians, including pharmacists. Clinicians can also ask questions via the smart medication dispenser and provide virtual clinical interventions as required.

### Outcome Measures

#### Adherence

Adherence data were determined by using the smart medication dispenser and AdhereNet, which receives data from the smart medication dispenser and displays adherence in real time, allowing for remote viewing and analysis by pharmacists. Adherence was measured by tracking the removal of a medication dose no more than 2 hours after the scheduled dosing time. For instance, if a patient’s medication was removed from the smart medication dispenser within this 2-hour window, the dose event was recorded as 100%. Conversely, if the dose was removed from the smart medication dispenser more than 2 hours after the scheduled time, the dose event was recorded as 0%. The daily medication adherence percentage was calculated based on the number of doses dispensed within a 2-hour window each 24-hour period. Monthly adherence reports were generated for each patient. The total average medication adherence for the study population (mean, SD) was calculated by dividing individual adherence percentage values over the course of the study by the number of study participants (N):




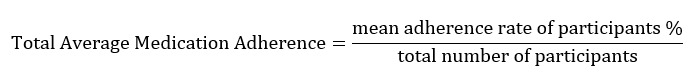




Daily medication intake data were collected for each participant using the smart medication dispenser. Mean monthly adherence was calculated at every 30-day interval (eg, period 1: 1-30 days, period 2: 31-60 days, period 3: 61-90 days, etc) for the duration of the study. Since participants started at different dates, we defined their first day using the smart medication dispenser as day 1. For any dates in which adherence data was not collected, the carry forward technique was utilized to impute these missing values [32]. This technique was applied to a maximum of 6 consecutive missing data points within a 30-day period. Participants with 7 or more consecutive missing data points were removed from that 30-day period of analysis.

#### Usability, Usefulness, and Satisfaction

Usability, usefulness, and satisfaction were measured with two tools: the System Usability Scale (SUS) and selected questions from the Usefulness, Satisfaction, and Ease of Use (USE) questionnaire. SUS is a broadly used 10-item posttest instrument that can be quickly administered to examine the usability of a product. Each item is scored on a 5-point Likert scale examining the degree of agreement. SUS has been demonstrated to be reliable and sensitive to successful task completion and identify differences in user experiences in multiple studies [33]. The USE questionnaire is a 30-item questionnaire designed to investigate the usefulness, ease-of use, ease of learning, and satisfaction of a system [34]. The 30 statements are rated on a 7-point Likert scale, ranging from strongly disagree to strongly agree. The questionnaire can be utilized over various assessments of usability, including both technology and nontechnology systems [34]. Although the questionnaire has not been studied among older adults, a psychometric evaluation among users of Microsoft Word and Amazon.com revealed a Cronbach alpha of .98 of the overall score. The USE questionnaire correlated well with SUS (*r* between 0.6 and 0.8) [35]. Due to the participant profile in this study, 9 statements regarding usefulness and satisfaction were selected out of the 30 total.

#### Pharmacist Clinical Interventions and Resolution of Drug Therapy Problems

Drug therapy problems were identified by pharmacists during their direct interactions with the participants. Direct interactions were conducted in person, over the phone, or through the smart medication dispenser’s telehealth video call functionality. Pharmacists interacted with patients to assess drug therapy problems at the beginning of the study, during the study as required, and at the end of the 6-month study period. The COVID-19 pandemic impacted pharmacists’ ability to conduct direct in-person interactions during the latter part of the study, resulting in most interactions occurring over the phone or over the smart medication dispenser’s telehealth video calling functionality. A drug therapy problem was defined as “any undesirable event or risk experienced by the patient that involves or is suspected to involve drug therapy and that interferes with achieving the desired goals of therapy and requires professional judgment to resolve” [36]. Drug therapy problems were classified based on the Canadian Consensus of Clinical Pharmacy Key Performance Indicators [36].

#### Caregiver Impact

Caregiver burden was measured on a visual analog scale by asking participants to indicate their response to the question, “On a scale of 0 to 10, how would you rate your burden level with respect to managing your loved one’s medications, where 0 = no burden and 10 = most burden?” Caregiver burden was measured at baseline and at the end of the 6-month study period.

### Statistical Analysis

Data were entered in a Microsoft Excel spreadsheet (Microsoft 365, version 2170; Microsoft Corp). Descriptive statistics (mean, standard deviation, and frequencies) were analyzed using Microsoft Excel. Independent *t* tests and Pearson chi-square tests were conducted using RStudio (1.2.1335) to examine if there was a statistically significant difference between participants who completed the study and those who dropped out. A paired *t* test was also conducted using RStudio to examine if there was a statistically significant change in caregiver burden from baseline to the end of the 6-month study period. Descriptive statistics were used to describe identified drug therapy problems.

### Ethics Approval

This study was reviewed by and received ethical approval from the University of Waterloo Office of Research Ethics (#40820). All participants were informed of the study and provided consent before enrolling.

## Results

### Participant Demographics

A total of 58 participants were recruited for this study, of whom 1 participant died and 9 participants withdrew consent prior to the end of the study but did not withdraw their data collected prior to their discontinuation in this study. Table 1 outlines the participants’ demographic statistics.

Of the 11 caregiver participants recruited, 91% (10/11) were female with a mean age of 57 (SD 16.6; range 28-83) years. There were no caregiver participant dropouts. Table 2 outlines the caregiver participants’ demographic statistics.

Almost all caregiver participants noted forgetfulness (n=9, 81.82%) or administering medications inappropriately (n=6, 54.55%) as reasons for starting to help their family members with their medications.

**Table 1:** Demographic characteristics of participants.

Variable		Total (N=58)	Withdrawn (n=9)	Completed (n=49)	*P* value	
**Age (years)**						.48 (*t*_56_=0.70935, CI −5.58 to 11.70)^a^
	Mean	66.36	69.56	65.78		
	SD	11.28	5.92	11.96		
	Mode	64	64	64		
	Median	65.5	69	64		
	Min	48	62	48		
	Max	90	80	90		
**Gender** **, n (%)**						.65 (*χ*^2^_1_=0.20147)^b^
	Male	26 (44.83)	3 (33.33)	23 (46.94)		
	Female	32 (55.17)	6 (66.67)	26 (53.06)		
**Marital status, n (%)**						.48 (*χ*^2^_5_=4.4642)^b^
	Single	8 (13.79)	1 (11.11)	7 (14.29)		
	Married	25 (43.10)	2 (22.22)	23 (46.94)		
	Widowed	9 (15.52)	1 (11.11)	8 (16.33)		
	Divorced	9 (15.52)	3 (33.33)	6 (12.24)		
	Separated	4 (6.90)	0 (0)	4 (8.16)		
	Living together	2 (3.45)	1 (11.11)	1 (2.04)		
	No response	0 (0)	1 (11.11)	0 (0)		
**Living arrangement, n (%)**						.16 (*χ*^2^_4_=6.5325)^b^
	Lives alone	22 (37.93)	4 (44.44)	18 (36.73)		
	With spouse	25 (43.10)	3 (33.33)	22 (44.90)		
	With partner	1 (1.72)	0 (0)	1 (2.04)		
	With family	9 (15.52)	1 (11.11)	8 (16.33)		
	Other	1 (1.72)	1 (11.11)	0 (0)		
**Ask for help with taking medications, n (%)**						.34 (*χ*^2^_1_=0.90484)^b^
	Yes	14 (24.14)	3 (33.33)	11 (22.45)		
	No	44 (75.86)	6 (66.67)	38 (77.55)		
**For participants who ask for help with taking medications, who helps?, n (%)**						
	Family member	14 (100)	3 (100)	11 (100)		
	Neighbor	0 (0)	0 (0)	0 (0)		
	Friend	0 (0)	0 (0)	0 (0)		
	Paid caregiver	0 (0)	0 (0)	0 (0)		
**Do you use a medication management aid?, n (%)**						.22 (*χ*^2^_1_=1.4895)^b^
	Yes	40 (68.97)	8 (88.89)	32 (65.31)		
	No	18 (31.03)	1 (11.11)	17 (34.69)		

^a^Independent 2-sample *t* test was performed.

^b^Pearson chi-square test was performed.

**Table 2:** Demographic characteristics of caregiver participants (n=11).

Characteristics				Values
**Age (years)**				
	Mean		57	
	SD		16.58	
	Mode		28	
	Median		59	
	Min		28	
	Max		83	
**Gender, n (%)**				
	Male		1 (9.09)	
	Female		10 (0.91)	
**Relationship, n (%)**				
	Family member		11 (100)	
**How often do you provide help?, n (%)**				
	>Once daily		7 (63.64)	
	Once daily		1 (9.09)	
	Once a week		2 (18.18)	
	≥Once a month		1 (9.09)	
**Have you ever provided a medication taking aid?, n (%)**				
	**Yes**		8 (72.73)	
		Blister pack	3 (37.50)	
		Blister pack and reminder	2 (25.00)	
		Dosette and reminder	2 (25.00)	
		Blister pack, dosette, and dispenser with alarm	1 (12.50)	
	No		3 (27.27)	

### Adherence

**Figure 1:**
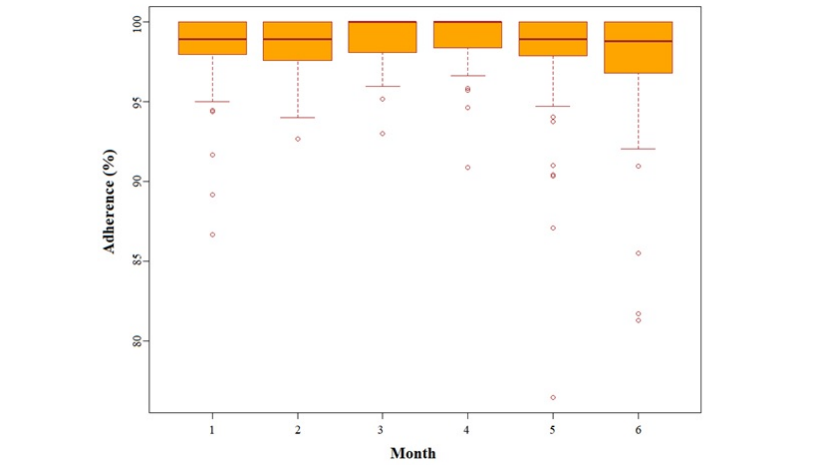
Monthly adherence.

The average monthly adherence over 6 months was 98% (n=56; SD 3.1%; range 76.5%-100%) (see Figure 1).

### Usability, Usefulness, and Satisfaction

**Figure 2:**
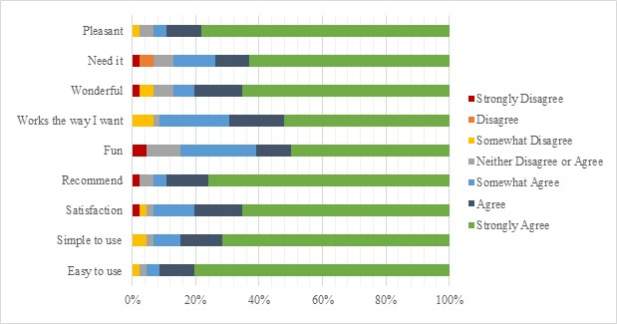
Usefulness, Satisfaction, and Ease of Use (USE) questionnaire response breakdown.

A total of 47 participants completed the SUS. The average SUS score was 85.74 (SD 12.7; range: 47.5-100). Meanwhile, 46 participants completed the 9-question USE questionnaire. Most participants (≥75%) strongly agreed that the smart medication dispenser was pleasant and easy to use (see Figure 2). Of the participants who completed the USE questionnaire, 43 (93%) indicated that they either somewhat agreed, agreed, or strongly agreed that they were satisfied with the product and would recommend the smart medication dispenser to a friend. Moreover, 42 (91%) participants somewhat agreed, agreed, or strongly agreed with the statement that they found the smart medication dispenser worked the way they want it to, and 40 (87%) somewhat agreed, agreed, or strongly agreed with the statement that they needed to have the product.

### Pharmacist Clinical Interventions and Resolution of Drug Therapy Problems

Drug therapy problems were identified at the time of the participant’s medication review and throughout the study time frame as part of routine practice. A total of 117 drug therapy problems were identified during the study period (see Table 3). These drug therapy problems affected 39 of the participants. Participants had on average 3 drug therapy problems (SD 2.08; range 1-9). Drug therapy problems occurred with 68 unique medications, and the most common medications with which problems were reported were vitamin D (15%), pantoprazole (7%), and acetaminophen (4%).

Drug therapy problems were most frequently reported with over-the-counter medications (42.74%) and most frequently classified as a need for a medication to be initiated (40.17%). Pharmacists most frequently requested an initiation (41.03%) or a discontinuation (22.22%) of medications.

**Table 3:** Drug therapy problems (N=117).

Drug therapy problems			Values, n (%)
**Type of medication**			
	Prescription high alert (Institute for Safe Medication Practices definition)^a^	19 (16.24)	
	Prescription nonhigh alert	48 (41.03)	
	Over the counter	50 (42.74)	
**Drug therapy problem classification**			
	Therapeutic duplication	2 (1.71)	
	Requires drug	47 (40.17)	
	Suboptimal response to a drug	10 (8.55)	
	Dosage is too low	4 (3.42)	
	Adverse drug reaction	19 (16.24)	
	Dangerously high dose	23 (19.66)	
	Noncompliance	3 (2.56)	
	Prescription has been confirmed false or has been altered	0 (0)	
	Other	11 (9.40)	
**Pharmacist recommendations**			
	Discontinue medication	26 (22.22)	
	Start medication	48 (41.08)	
	Start alternative nonpharmaceutical therapy	3 (2.56)	
	Change dose	16 (13.68)	
	Change route	0 (0)	
	Change schedule	10 (8.55)	
	Dosage strength	0 (0)	
	Change dosage form	3 (2.56)	
	Change duration of treatment	1 (0.85)	
	Recommend monitoring	8 (6.84)	
	Provide patient education	7 (5.98)	
	Continue medication	1 (0.85)	
	Refer to a physician or nurse practitioner	5 (4.27)	
**Drug therapy problem follow-up/resolution**			
	Problem resolved: recommendation accepted by patient	23 (19.66)	
	Problem resolved: recommendation accepted by physician or nurse practitioner	6 (5.13)	
	Problem unresolved: recommendation not accepted by patient or prescriber	1 (0.85)	

^a^Institute of Safe Medication Practices defines prescription high alert medications as “drugs that bear a heightened risk of causing significant patient harm when they are used in error” [37].

### Caregiver Burden Impact

Caregiver burden scores were obtained from 11 caregivers before and following the use of the smart medication dispenser for 6 months. The average caregiver burden scores at baseline were 7/10 (SD 2.6; range 1-10) and 3/10 (SD 2.8; range 0-8) following product use, which were found to be statistically significantly different (*P<*.001; CI 2.11-5.98).

## Discussion

### Principal Findings

Many factors impact adherence to medications, including patient-related factors, medication-related factors, social and economic factors, and health care–related factors. Among patient-related factors, unintentional medication nonadherence may arise due to forgetfulness and physical and cognitive limitations [1]. Among medication-related factors, nonadherence may be related to multiple or complex medication regimens [1]. Automated dispensing devices may help improve adherence in such circumstances. However, the usability and effectiveness of these devices in addressing medication nonadherence need to be further examined. Our study investigated the integration of a smart multidose medication dispensing aid with real-time medication intake monitoring capacity in the home of adults taking chronic medications. Over 6 months, participants had a mean adherence rate of 98% with the use of the smart medication dispenser. The SUS score for usability was 85.74, and >75% of the participants found the product pleasant and easy to use. Finally, the integration of the smart medication dispenser into the home significantly decreased caregiver burden and provided a space for pharmacists to conduct medication reviews and identify drug therapy problems.

A total of 117 drug therapy problems were identified in 39 participants; 20% of the recommended changes to address the drug related problems were accepted by the patient, 5% were accepted by the physician or nurse practitioner, and 75% remained unresolved (1%) or not reported (74%). Unfortunately, the timing of this study intersected with the COVID-19 pandemic, which impacted the ability of pharmacists to reach prescribers and obtain follow-up on recommended changes to address drug-related problems. Most physician offices were closed or offered limited services, which impacted communication between physicians and pharmacists.

Communication between pharmacists and patients, however, was not hampered due to the availability of telephone and video calling using the smart medication dispenser. While we did not intend to track patient preference for these services, we found that participants preferred telephone calling to video calling. This coincides with the findings reported by Rodriguez et al [38]. In their study, participants who were 65 years of age or older were less likely to use video visits compared with those aged 18-64 years [38]. Similarly, in a study describing the transition to telemedicine in the geriatric primary care population, Schifeling et al [39] reported that more than half of the patients used telephone visits. Future studies should further investigate preference and rationale for preference in telemedicine care (telephone versus video calling) and its impact, if any, on patient satisfaction and the quality of care received. Older adults may prefer interactions through telephone as it is a familiar, accessible, and easy to use mechanism of communicating. Video calling, even if enabled through medication dispensing technology, requires one to learn how to use the system, impacting its usability and uptake. Other studies have reported similar findings [38,39].

The monthly adherence rate in this observational study remained >95% over the 6 months. Our study was not designed to investigate the effectiveness of the smart medication dispenser in improving adherence. As such, we did not seek to enroll participants deemed to be nonadherent to their medications, nor did we capture medication adherence rates at baseline to permit a comparison at the end of the 6-month study period. We also deemed a participant to be adherent to their medications if they retrieved the multidose package dispensed to them within a 2-hour time frame following their scheduled medication dosing time. Other smart dispensing devices have assessed adherence in a variety of patient populations; however, in these studies, there is significant variability in the definition and measurement of adherence. Where adherence rate was determined by the use of a smart dispensing device, rates of reported adherence ranged from 93% to 97% [21]. Additionally, previous studies have demonstrated that electronic drug monitors accurately measure times of opening of pill bottles in nonclinical settings and improve adherence [11,40-42]. Furthermore, electronic drug monitoring is widely regarded as the gold standard for measuring adherence [43] and provides an insight into medication taking behavior in the home, which in turn may help to identify specific challenges patients may encounter when taking their medications in the home. For example, if patients consistently fail to retrieve the medication pouches for a particular dosing time, clinicians can design strategies to address this challenge. If a dosing schedule can be altered to meet the needs of the patient’s lifestyle or needs, this can then be implemented. In this way, this system encourages a patient-centered approach to medication taking and the measurement of adherence. Furthermore, a review of medication intake data and feedback to patients can help uncover other contributors to nonadherence such as medication adverse effects and beliefs about the need for medications, among others [42]. In 2013, Demonceau et al [44] determined that adherence feedback to patients based on drug dosing histories improved adherence by 8.8%. More recently, van Heuckelum et al [45] determined that electronic medication feedback had a significantly positive effect on medication adherence.

We examined the usability of a smart medication dispenser in the home using SUS. Field testing of usability in the home is rarely conducted [19,46], and while there are other methods of testing the usability, the simplicity and ease of use of a 10-item questionnaire addressed the need for a practical measure. Furthermore, SUS is the tool most frequently used to measure the usability of products [33]. In this study, the mean SUS score of the medication dispensing device was 85.74. While there are no benchmarks against which to compare the SUS score, one study examined the usability of 21 electronic medication adherence products with SUS [47]. Although most of the medication dispensing aids tested in the study were not smart, and only one permitted electronic monitoring of medication dispensing, many of the dispensing aids had typical features of dispensing aids used by patients. In that study, SUS scores ranged from 28.63 to 78.67, and the mean score was 52.28 [47]. In comparison to that score, the SUS score for this smart medication dispenser was substantially higher, indicating higher usability of the product. Furthermore, most participants reported the smart medication dispenser was easy and simple to use and were satisfied with the product, potentially predicting ease of implementation in the home by patients, in particular older adults.

Maintaining medication adherence is complex. When the capacity to manage medications declines, unpaid caregivers such as family members often step in to assist patients with managing their medications. Results from our study demonstrate a significant decline in the visual analog scale score for burden associated with managing medications from baseline to the end of the study period. In their study, Polenick et al [29] demonstrated that medication reminder systems decreased caregiver burden among caregivers of persons with dementia but not among those care giving for persons without dementia. However, in our study, people with cognitive impairment were excluded. Several aspects of the smart medication dispenser may have contributed to the decline in caregiver burden in our study population. The administration of prepackaged medications in pouches at appropriate times alleviates the caregiver’s need to organize and administer the medications. Additionally, caregivers who do not reside with the patient no longer have to schedule multiple visits throughout the day to ensure medications are taken as appropriate, as medication taking can be confirmed virtually through medication intake monitoring.

### Limitations

Although the caregiver burden decreased significantly over the study duration, this is a hypothesis generating finding, as this study was not powered to investigate caregiver burden as a primary objective. Furthermore, we utilized only one question on a visual analog scale to examine the impact of the smart medication dispenser on caregiving burden. This question does not provide an insight into the aspects of caregiving related to medication management that the use of the smart medication dispenser produces. However, the currently available tools do not specifically measure caregiving burden related to the management of medications [48]. The Family Caregiver Medication Administration Hassles tool permits the examination of strain related to medication management but consists of domains pertaining to monitoring the safety of medications as well as scheduling and administration issues. Some components of this tool could be utilized to measure caregiver strain related to medication management and should be considered in future studies [49].

Missing adherence data were addressed by utilizing the carry forward technique. Though this technique was used to account for incidents where adherence was not tracked by the smart medication dispenser even though the patient took their medication, it may have overrepresented the true rate of adherence. Medication adherence was not tracked when system or user error led to loss of data. Examples of such system errors include disconnecting the device from the internet, removing batteries from the device, or another indeterminate system error that led to data loss.

### Conclusion

This was a pilot study designed to examine the feasibility of integrating the smart medication dispenser into the homes of patients, test its usability, and explore whether caregiver burden was affected. This study was not designed to measure the effectiveness of the smart medication dispenser in these domains. The adherence was potentially driven by usability of the device, personalization of medication administration times, and decrease in caregiving burden. The results of this study can be utilized to design hypothesis testing studies in the future. In particular, given that usability scores were high, future studies can be designed to examine the impact on adherence in nonadherent populations. Similarly, the impact on caregiver stress and burnout should be further examined.

## Abbreviations

SUS: System Usability Scale
USE: Usefulness, Satisfaction, and Ease of Use questionnaire
WHO: World Health Organization
